# Redefining prognostication of de novo cytogenetically normal acute myeloid leukemia in young adults

**DOI:** 10.1038/s41408-020-00373-4

**Published:** 2020-10-19

**Authors:** Sze P. Tsui, Ho W. Ip, Nicole Y. Saw, Chunxiao Zhang, Arthur K. Cheung, Nelson K. Ng, Cheuk H. Man, Stephen S. Lam, Wing F. Tang, Chi H. Lin, Grace H. Cheng, Chun H. Au, Edmond S. Ma, Tsun L. Chan, Jason C. So, Margaret H. Ng, Kelvin C. Cheng, Kit F. Wong, Lai P. Siu, Sze F. Yip, Shek Y. Lin, June S. Lau, Tsan H. Luk, Harold K. Lee, Chi K. Lau, Bonnie Kho, Joycelyn P. Sim, Yok L. Kwong, Suet Y. Leung, Asif Javed, Anskar Y. Leung

**Affiliations:** 1 Department of Pathology, Queen Mary Hospital, Hong Kong SAR, China; 2 School of Biomedical Sciences, LKS Faculty of Medicine, The University of Hong Kong, Hong Kong SAR, China; 3 Division of Haematology, Department of Medicine, LKS Faculty of Medicine, The University of Hong Kong, Pokfulam, Hong Kong SAR, China; 4 Centre for PanorOmic Sciences, LKS Faculty of Medicine, The University of Hong Kong, Pokfulam, Hong Kong SAR, China; 5 The Jockey Club Centre for Clinical Innovation and Discovery, LKS Faculty of Medicine, The University of Hong Kong, Pokfulam, Hong Kong SAR, China; 6 Department of Pathology, Hong Kong Sanatorium and Hospital, Hong Kong SAR, China; 7 Department of Pathology, Hong Kong Children’s Hospital, Hong Kong SAR, China; 8 Department of Anatomical and Cellular Pathology, The Chinese University of Hong Kong, Hong Kong SAR, China; 9 Department of Pathology, Queen Elizabeth Hospital, Hong Kong SAR, China; 10 Department of Pathology, Tuen Mun Hospital, Hong Kong SAR, China; 11 Department of Medicine and Geriatrics, United Christian Hospital, Hong Kong SAR, China; 12 Department of Medicine, Queen Elizabeth Hospital, Hong Kong SAR, China; 13 Department of Medicine, Princess Margaret Hospital, Hong Kong SAR, China; 14 Department of Medicine, Tseung Kwan O Hospital, Hong Kong SAR, China; 15 Department of Medicine, Pamela Youde Nethersole Eastern Hospital, Hong Kong SAR, China; 16 Department of Pathology, LKS Faculty of Medicine, The University of Hong Kong, Pokfulam, Hong Kong SAR, China

**Keywords:** Acute myeloid leukaemia, Cancer genetics

Dear Editor,

About 50% of acute myeloid leukemia (AML) showed normal cytogenetics (CN) with leukemogenesis driven putatively by recurrent mutations. These mutations occur singly or in combination, as dominant clones or subclones^1–4^. Induction with daunorubicin and cytarabine is the standard for young and fit patients, achieving first complete remission (CR1) in 60–80% cases. Post-remission strategies include consolidation with high-dose cytarabine and allogeneic hematopoietic stem cell transplantation (allo-HSCT). The latter may reduce the risk of relapse but is associated with mortality and long-term morbidities. The European LeukemiaNet (ELN) guidelines, based on cytogenetic and genetic risk stratification, provide general recommendations on prognostication and allo-HSCT for AML^5^. We performed next-generation sequencing (NGS) for young patients with de novo CN-AML, diagnosed between 2003 and 2019, who were treated with a relatively uniform algorithm to examine the prognostic impact of mutation combinations. Machine learning was used to generate prediction model and its performance was compared with that based on ELN guidelines. Clinical treatment and methodology are described in Supplemental Materials (see also Supplemental Fig. S1).

Four hundred and fifty-nine patients with de novo CN-AML, at a median age of 49 years (range: 18–60 years), were studied (Supplemental Table S1). Their treatment outcomes are shown in Fig. 1A. Four hundred and thirty-six patients received induction chemotherapy, of whom 419 patients (96%) received standard “7 + 3” regimen and 17 (4%) received “5 + 2”, idarubicin, mitoxantrone, hypomethylating agents, or homoharringtonine-based regimens. After the first induction, CR/CRi (CR with incomplete hematological recovery) was achieved in 283 patients (65%). There was no significant difference in leukemia-free survival (LFS) (95% confidence interval (C.I.) 0.92–1.55; *P* = 0.18) or overall survival (OS) (95% C.I. 0.99–1.80; *P* = 0.06) between patients who achieved CR or CRi after first induction and they were analyzed together. High-dose daunorubicin (90 mg/m^2^) was associated with significantly higher chance of CR compared with standard dose (60 mg/m^2^; Supplemental Table S2). Patients who failed first induction received salvage chemotherapy (Supplemental Materials), resulting in CR1 in another 113 patients. Post-remission therapy included high-dose cytarabine, with some patients having received 1–2 courses of “5 + 2” before it. Allo-HSCT was performed in 181 patients from different donor types (HLA identical siblings, *N* = 103; matched unrelated, *N* = 75; haploidentical, *N* = 1; identical twins, *N* = 2).

**Fig. 1:**
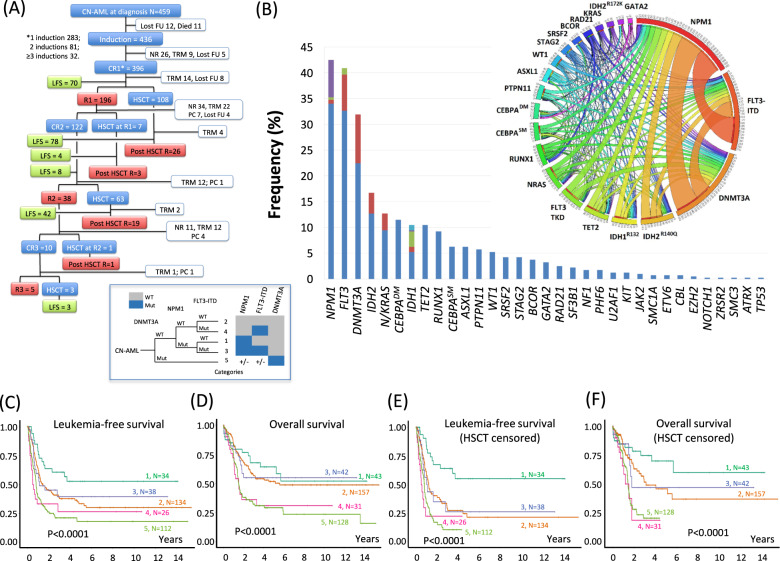
Clinical and mutational features and the prognostic impact of mutation combinations in cytogenetically normal acute myeloid leukemia (CN-AML). **A** Clinical outcome of 459 young patients (≤60 years) with CN-AML. FU follow-up, LFS leukemia-free survival, CR complete remission, NR non-remission, TRM treatment-related mortality, R relapse, PC palliative care, HSCT hematopoietic stem cell transplantation. **B** Mutation spectrum of 401 patients. Different mutations of each gene were shown in different colors. *NPM1*: Blue, Type A; Red, Type B; Green, Type D; Purple, other types; *FLT3*: Blue, ITD (Internal Tandem Duplication); Red, TKD (D835 mutations); Green, TKD (non D835 mutations); *DNMT3A*: Blue, R882 mutations; Red, non R882 mutations; *IDH2*: Blue, R140Q; Red, R172K; RAS: Blue, *NRAS*; Red, *KRAS*; *IDH1*: Blue, R132H, Red, R132S, Green, R132C, Purple, R132L, Aqua, R132G. Circos diagram demonstrated frequencies of co-occurrence of mutations. The width of the ribbons represented the number of patients with two co-existing mutations. The numbers indicated in the circumference exceeded the number of patients with that mutation as genes with multiple mutation partners were counted separately. **C**–**F** Leukemia-free (**C**, **E**) and overall survival (**D**, **F**) of patients with different mutations of *NPM1*, *FLT3*, and *DNMT3A*. **C**, **D** Patients with different mutation combinations, censored on 1 March 2019. **E**, **F** Survivals censored at hematopoietic stem cell transplantation. Leukemia-free survival was the duration from CR to the last follow-up, leukemia relapse, or death. Overall survival was the duration from diagnosis to last follow-up or death. Category 1: *NPM1* mutation only; Category 2: All wild type; Category 3: *NPM1* mutation and *FLT3*-ITD. Category 4: *FLT3*-ITD only; Category 5: *DNMT3A* mutation irrespective of *NPM1* and *FLT3* status. The insert defines the five categories.

For the initial 187 patients analyzed by the pan-cancer panel (Supplemental Data 1), mutations were identified in 77 genes, with 42 genes mutated in ≥1% and 13 genes mutated in ≥5% of patients (median: 3 mutations per patient; range: 0–7). Subsequently, 43 patients were analyzed by the myeloid-focused panel, 33 patients by the Trusight panel, and 138 patients by the AML panel. In these 214 patients, mutations were identified in 29 genes (median: 3 mutations per patient; range: 0–6). Mutations were categorized according to their putative functions in hematopoiesis or leukemogenesis (Supplemental Table S3). The most frequently mutated genes are shown in Fig. 1B. *NPM1*, *DNMT3A*, *CEBPA*^DM^, and *IDH1/2* often showed variant allele frequency (VAF) of 40–50%; whereas *FLT3*-ITD, *NRAS*, and *FLT3*-TKD showed more heterogeneous VAF of 10–50% (Supplemental Fig. S2).

*NPM1* and *CEBPA*^DM^ mutations were associated with superior CR/CRi rates and *RUNX1* and *ASXL1* mutations with inferior CR/CRi rates after first induction (Supplemental Table S4). To examine the factors affecting survivals, age, gender, white blood cell count (WCC), daunorubicin dose (60 versus 90 mg/m^2^), achievement of CR/CRi after induction or salvage chemotherapy, allo-HSCT at CR1 as well as individual gene mutations were analyzed by univariate analysis. Age and WCC varied with LFS, event-free survival (EFS), and OS as continuous functions and were defined as numerical data (Supplemental Fig. S3). High-dose daunorubicin and HSCT at CR1 were associated with superior LFS, EFS, and OS and achievement of CR/CRi was associated with superior EFS and OS, whereas high WCC and *FLT3*-ITD and *DNMT3A* mutations were associated with inferior LFS, EFS, and OS (Supplemental Table S5A). High-dose daunorubicin appeared to negate the adverse prognosis of *DNMT3A* mutations, consistent with previous reports^6^ (Supplemental Fig. S4). Subsequently, these factors were evaluated in multivariate analysis. The prognostic impacts of *FLT3*-ITD and *DNMT3A* mutations, achievement of CR/CRi, and HSCT at CR1 have remained unchanged but those of high-dose daunorubicin have become largely insignificant (Supplemental Table S5B). *NPM1* mutation was associated with superior LFS and EFS but not OS.

*NPM1*, *DNMT3A*, and *FLT3*-ITD were further evaluated for their relative impacts on LFS (Supplemental Fig. S5) and OS (Supplemental Fig. S6). *DNMT3A* mutation negated the prognostic impact of *NPM1* mutation and *FLT3*-ITD, attesting to its overriding impact on prognosis amidst co-existing mutations. *FLT3*-ITD also negated the prognostic impact of *NPM1* but not *DNMT3A* mutation. *NPM1* mutation had no significant impact on the adverse prognostic effects of *DNMT3A* mutation and *FLT3*-ITD. Their combinations showed variable LFS and OS (Supplemental Fig. S7) and were further categorized into five groups (Supplemental Table S6). Sole *NPM1* mutation (Category 1) showed superior LFS and OS while sole *FLT3*-ITD (Category 4) and presence of *DNMT3A* mutation (Category 5) showed inferior LFS and OS. Patients of wild type for all 3 genes (Category 2) and with co-existing NPM1 mutation and *FLT3*-ITD (Category 3) showed intermediate LFS. However, their OS were indistinguishable from that of Category 1 (Fig. 1C, D). When outcomes were censored at HSCT, Category 1 remained superior, Categories 2 and 3 were intermediate, and Categories 4 and 5 remained inferior (Fig. 1E, F). Subgroup analyses were performed to evaluate the prognostic impact of other recurrent mutations on the five categories. *IDH1*R132H was associated with inferior LFS and OS in Category 2 exclusively (Supplemental Fig. S8). Other mutations had no significant impact on these categories or their occurrences were too low for comparison (Supplemental Table S7).

To examine whether prognostication by ELN 2017 guidelines might apply to young patients with CN-AML, the present cohort was classified according to the stipulated risk groups, based exclusively on gene mutations. High FLT3-ITD was defined by VAF ≥0.33, corresponding to an allelic ratio of ≥0.5 (Supplemental Table S8). There was a trend toward a difference in LFS and OS among the three risk groups. However, it was statistically insignificant (Fig. 2A, B). We examined the impact of *DNMT3A* mutation on each ELN-defined risk groups in our patients. *DNMT3A* mutation negatively impacted on LFS and OS in the favorable (Supplemental Fig. S9A, B) and intermediate risk groups (Supplemental Fig. S9C, D) but not in the unfavorable risk group (Supplemental Fig. S9E, F). Incorporating *DNMT3A* mutation into the ELN risk categorization as an unfavorable risk factor separated the three risk groups and significantly improved the risk stratification (Fig. 2C, D).

**Fig. 2:**
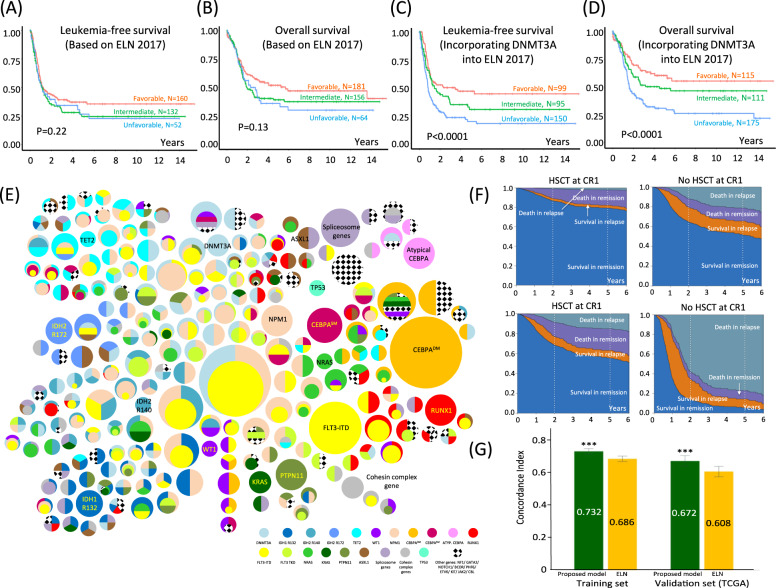
Patient outcome based on European LeukemiaNet (ELN), clonal heterogeneity, and prediction model of cytogenetically normal acute myeloid leukemia. **A**, **B** Leukemia-free survival (**A**) and overall survival (**B**) according to ELN 2017 guidelines. **C**, **D** Leukemia-free survival (**C**) and overall survival (**D**) after incorporating *DNMT3A* mutation as unfavorable risk group. Leukemia-free survival was the duration from CR to the last follow-up, leukemia relapse, or death. Overall survival was the duration from diagnosis to last follow-up or death. **E** A bubble diagram showing clonal heterogeneity in CN-AML. Each mutation was represented by a distinct color except the checker that represented any of the rare mutations as shown. The size of each outer bubble (dominant or co-dominant) indicated the prevalence of patients with that genotype. Inner bubble indicated subclone and its size represented the clone size relative to that of the dominant or co-dominant clones. Horizontal bisection of inner bubbles indicated occurrence of either one of the mutations, whereas vertical bisection indicated occurrence of both mutations. **F** Sediment plots of two hypothetical patients who received allogeneic HSCT at first complete remission (CR1) or not, based on prediction model using machine learning of the present cohort of patients. The shaded areas indicated the time courses of different outcomes. Upper panels: A 25-year-old female patient presenting with white cell counts of 10 × 10^9^/L and genotype category 1 (*NPM1* mutation only) who achieved CR1 after first induction and is considering allo-HSCT at CR1. Her chances of leukemia-free survival would be 87% at 2 years and 79% at 5 years post HSCT. If she declines HSCT, the chances would be reduced to 64% at 2 years and 52% at 5 years. Lower panels: A 25-year-old female patient presenting with white cell counts of 100 × 10^9^/L and genotype category 5 (*DNMT3A* mutation) is considering allo-HSCT at CR1. Her chances of leukemia-free survival would be 68% at 2 years and 55% at 5 years. Should this patient decline HSCT, her chances of surviving the leukemia would become 14% at 2 years and only 5% at 5 years with a 77% likelihood of death in relapse. **G** Histogram showing concordance index in the present cohort (training set) and a cohort of young patients (≤60 years old) with CN-AML in the TCGA cohort (validation set). The green bars indicated results from the reported prediction model and the yellow bars represented results if we categorize patients based on ELN 2017 risk stratification. The error bars indicated a standard error of mean. ***P < 0.001.

The genetic makeup of leukemic clones was extremely diverse (Fig. 2E and Supplemental Fig. S10). Of the 401 patients on whom NGS was performed, 383 patients showed detectable mutation of genes in the AML panel with at least 217 distinct clonal subtypes. The most common subtypes comprised co-dominant *NPM1* and *DNMT3A* mutations, usually in conjunction with other co-dominant or subclone mutations. Sole *NPM1* (1.00%) or *DNMT3A* mutations (0.50%) or their co-dominance without subclones (0.50%) were relatively uncommon. *NPM1* mutation was infrequently found in subclones, and in those rare circumstances, the dominant clones were mostly *DNMT3A* or *IDH2*R140Q mutations. *FLT3*-ITD occurred most frequently as subclones. However, in 2.74% patients, *FLT3*-ITD occurred as the sole mutation, suggesting its role as leukemic driver early in the leukemic hierarchy^7^. *CEBPA*^DM^ occurred predominantly as a sole mutation in 5.24% patients. Forty-six patients (11.47%) were negative for all common or ELN risk-defining mutations, viz. *NPM1*, *DNMT3A*, *FLT3*, *IDH1/2*, *CEBPA*, *ASXL1*, *RUNX1*, and *TP53*. They showed rare mutations, some of which, including those of spliceosome genes^8^, were dominant and sole mutations, suggesting pathogenetic role in leukemogenesis (Supplemental Materials, Supplemental Fig. S11, and Supplemental Table S9).

The database built up in this study formed a foundation for the development of prediction model (https://redefiningprognosis.shinyapps.io/denovo_cnaml/) that might inform clinical decision. Its application was highlighted by two hypothetical patients (Fig. 2F). The information provided quantitative measurement of survival benefits of individual patients based on their demographics and genotypes. Its performance was compared with that of the ELN risk stratification-based model based on concordance index. Using the present cohort of 401 patients as a training set, our prediction model showed a 4.6% higher concordance over the ELN-based model (Fig. 2G). To validate these models, a subset of The Cancer Genome Atlas patients comprising 83 de novo CN-AML patients aged ≤60 years was used as a validation cohort. Patients who received HSCT at refractory stage were not included as they were not represented in the training set. Again, our model showed 6.45% higher concordance over ELN-based model. The difference in concordance in both cohorts was statistically significant. We proposed that this multistage model might provide more personalized guidance to inform post-remission therapy with particular reference to allo-HSCT^9^. Our findings corroborated with recent reports demonstrating room to refine risk stratification based on sequencing and transcriptomic results of patients enrolled into clinical trials^10^.

In conclusion, in young patients with de novo CN-AML who received conventional induction chemotherapy, consolidation, and allo-HSCT, incorporation of *DNMT3A* mutation into risk stratification significantly improved their prognostication. Predication model based on machine learning of our database generated a more personalized tool to guide post-remission therapy. The diverse clonal heterogeneity and the pathogenetic significance of mutation combinations provided important leads for future mechanistic study.

## Supplementary information

Supplemental Materials

Supplemental figure S1

Supplemental figure S2

Supplemental figure S3

Supplemental figure S4

Supplemental figure S5

Supplemental figure S6

Supplemental figure S7

Supplemental figure S8

Supplemental figure S9

Supplemental figure S10

Supplemental figure S11

Supplemental tables

Dataset 1

## Data Availability

The datasets generated and analyzed during the current study are available from the corresponding author on reasonable request.
