# Supplementary material for: Redefining prognostication of de novo cytogenetically normal acute myeloid leukemia in young adults
Source: Blood Cancer J. 2020 Oct 19;10(10):104. doi: 10.1038/s41408-020-00373-4 (PMC7573626; doi:10.1038/s41408-020-00373-4)
Supplement: Supplementary file 1 — Supplemental Materials [file 41408_2020_373_MOESM1_ESM.docx]

**Supplemental Materials**

*Treatment algorithms*

Induction comprised daunorubicin 60 or 90 mg/m^2^ for three days and cytarabine 100 mg/m^2^ for seven days (7 + 3 regimen) and in the minority of cases other regimens. Bone marrow (BM) assessment was performed at the time of count recovery or sign of non-remission at physicians’ discretion. Complete remission (CR) was defined by the absence of blasts (≤5%) in BM and complete hematologic recovery (absolute neutrophil count ≥ 1x10^9^/L and platelet count ≥ 100 x 10^9^/L). CRi was defined as CR with incomplete hematologic recovery (absolute neutrophil count < 1x10^9^/L or platelet count < 100 x 10^9^/L). Consolidation comprised high dose cytarabine 3 gm/m^2^ for 4-6 doses for 3-4 cycles (HDAC). Some patients received additional “5+2” regimen, comprising daunorubicin 50 mg/m^2^ for two days and cytarabine 100 mg/m^2^ for five days as consolidation. All patients received antifungal prophylaxis and G-CSF (Granulocyte-Colony Stimulating Factor) during neutropenia. Patients who did not achieve CR after first induction received salvage chemotherapy.

Salvage chemotherapy regimens used in patients who failed 7+3 induction.

| Chemotherapy regimens | N |
| --- | --- |
| Mitoxantrone and intermediate dose cytarabine (MAC) | 36 |
| Idarubicin, cytarabine and etoposide (ICE) | 27 |
| Fludarabine, cytarabine and G-CSF with or without idarubicin (FLAG ± Ida) | 22 |
| Clofarabine based regimens | 8 |
| Daunorubicin and cytarabine (second course) | 28 |
| Others | 12 |

N: Number of patients receiving salvage regimens. Patients might have multiple entries if they have received multiple salvage regimens and those who received multiple courses of a particular regimen would have only one entry. Others: FLT3 inhibitors and homohorringtonine

From 2009, non-remission patients with internal tandem duplication of the *FLT3* gene (*FLT3*-ITD) received *FLT3* inhibitor as part of the salvage therapy.^1-3^ All transplant-eligible patients were referred to Queen Mary Hospital, Hong Kong, for consideration of allo-HSCT. Indications for allo-HSCT in CN-AML at CR1 included hyperleukocytosis (white cell count, WCC≥ 100 x 10^9^/L), central nervous system (CNS) infiltration, extramedullary disease or *FLT3*-ITD at diagnosis. Patients who received two courses of chemotherapy to achieve remission or who relapsed and achieved second remission (CR2) with salvage chemotherapy were also considered for allo-HSCT.

Salvage chemotherapy regimens used in relapsed cases

| Chemotherapy regimens | N |
| --- | --- |
| Mitoxantrone and intermediate dose cytarabine (MAC) | 36 |
| Idarubicin, cytarabine and etoposide (ICE) | 24 |
| Fludarabine, cytarabine and G-CSF with or without idarubicin (FLAG ± Ida) | 27 |
| Clofarabine based regimens | 23 |
| Daunorubicin and cytarabine | 7 |
| Others | 24 |

N: Number of patients receiving salvage regimens at relapse. Patients might have multiple entries if they have received multiple salvage regimens and those who received multiple courses of a particular regimen would have only one entry. Others: Hypomethylating agents, amsacrine-based, homoharringtonine-based regimens, FLT3 inhibitors.

The study was approved by Institutional Review Boards of Hospital Authority [HKU/HA HKW UW14-430 and UW14-639; KC/KE--15-0039/ER-3; KW/EX-15-052/85-05; NTWC/CREC/15013; KCKE-15-0098(ER-1) and HKEC-2015-005].

*Next generation sequencing (NGS) and bioinformatics analyses*

Genomic DNA was extracted from diagnostic BM (N=374) or peripheral blood (PB) (N=18). Relapsed BM samples were used in nine patients whose diagnostic samples were unavailable. NGS was performed using three different targeted capture panels. Initially a pan-cancer panel comprising 544 recurrent mutations in malignancies was used. Subsequently, three more focused gene panels targeting 67 genes (myeloid-focused panel), 54 genes (Trusight myeloid panel) and 36 genes (AML panel) were used (xGen Lockdown Probes, Integrated DNA Technologies, Coralville, Iowa, USA) (Supplemental Data 1). DNA libraries were prepared using KAPA HyperPlus kit (Roche, Pleasanton, CA, USA) or sonication and KAPA HyperPrep kit (Roche, Pleasanton, CA, USA). Targeted capture of DNA libraries was performed and sequenced with either NovaSeq (Illumina, San Diego, CA, USA) for pan cancer panel, MiniSeq (Illumina, San Diego, CA, USA) for myeloid-focused and AML panels and MiSeq for Trusight myeloid panels. The sequencing depth of each samples were at least 400X.

Sequencing reads were processed to prepare alignment files according to the recommendation by Genome Analysis Toolkit (GATK) (version 3·8 for pan-cancer and myeloid-focused panels and version 3·7 for AML panel). A bioinformatic tool Cutadapt was used to trim the adapter sequence and remove low quality reads. The remaining reads were aligned to Human Genome version 19 (hg19) with a software package Burrows Wheeler-Alignment – Maximal Exact Matches (BWA-MEM) and sorted with a toolkit PICARD, which was also used to identify duplicate reads. GATK was used to realign reads around putative indel sites and recalibrate base quality. HaplotypeCaller was used to call variant sites. Variant quality score was recalibrated using VQSR. Variants that passed the recalibration were annotated using ANNOVAR. Pindel was used to identify *FLT3*-ITD. Variant call was initially restricted to coding regions and subsequently extended to include splice altering mutations. Known polymorphic sites (minor allele frequency ≥ 0·001), previously reported in the 1000 Genomes project, ExAC, Kaviar genomic, or haplotype reference consortium, were excluded. Further screening was conducted against an in-house dataset of 0·2M variant sites based on data from exome sequencing and a panel of 95 non-tumor gastric tissue samples from local patients sequenced by the pan-cancer panel.^4^ Multi-allelic indel sites with high likelihood of false positivity were removed.^4^ Bioinformatic tools used for Trusight Myeloid panel have been described previously.^5^ Results from different analytic pipelines were compared to ensure consistency and reproducibility. Filtered variants were analyzed manually with reference to the COSMIC or ClinVar databases.^6,7^ Frame-shift and nonsense variants were considered pathogenic whereas synonymous variants were considered benign unless there was splice site gain or loss. Unreported variants were analyzed *in silico* based on prediction softwares (SIFT, PolyPhen-2, PROVEAN and CADD) and damaging variants were identified.

Hierarchy of leukaemic clones in each patient sample was ascertained by comparing their variant allelic frequencies (VAF). VAF of homozygous gene mutation and those of mutant genes located on X-chromosome in male patients were reduced by 50%. For each patient, gene mutation with the highest VAF was defined as the dominant clone. VAF of different mutations in each patient were compared using the two-proportion z test, taking into consideration the absolute number of sequencing reads for each gene. Gene mutations in descending order of VAF defined the dominant and subsequent subclones in the hierarchical evolution.

*Development of prediction model based on machine learning*

To develop prediction model, the entire cohort was evaluated by a statistic package known as mstate, which analyzed competing risks at multiple stages during the course of disease (See Legends, Supplemental Figure S1).^8^ A concordance index (c-index) was calculated using the ‘*concordance*’ function in *survival* package in R to evaluate performance of each model. ^9,10^ Briefly, each patient had an actual OS and a predicted OS based on the specific model. The difference in OS between any given patient pair as predicted by this model was compared with that of their actual OS. The two patients were defined as concordant if the difference in predicted OS was supported by a similar difference in their actual OS. Concordance index was defined by the fraction of patient pairs that were concordant in all comparable pairs.

*Clonal architecture and its diversity* – *Rare mutations*

Forty-six patients (11·47%) were negative for all common or ELN risk defining mutations including *NPM1,* *DNMT3A*, *FLT3*, *IDH1/2*, *CEBPA*, *ASXL1*, *RUNX1* and *TP53*. Twenty-eight (6·98%) of them were positive for infrequent mutations previously described in myeloid neoplasms (Supplemental Figure S10). Specifically, mutations of spliceosome components occurred in nine patients (*SF3B1*=5; *U2AF1*=3 and *SRSF2*=1). These mutations were dominant in eight patients and were the sole mutations in six of them. Mutation of *PTPN11* occurred in five patients, being the dominant and sole mutations in four patients. Mutations of cohesin complex including *STAG2*, *RAD21*, *SMC1A*, occurred in four patients. They occurred as sole mutations in two patients, co-dominant mutation in one patient and subclone in one patient. Eighteen patients (4·49%) showed no mutation in the AML panel of whom 17 were also sequenced by the pan-cancer panel. Pathogenic mutations were identified in five patients (Supplemental Table S9), including those of combined *CUL4A*, *IGFN1*, *KMT2D* mutations in one patient and single *SBDS*, *CCND3*, *RAD54L*, *SETD2* mutation in four patients. Twelve patients had no cancer related mutations identified.

*Statistical analyses*

Continuous data were compared by unpaired Student’s t-test and Mann-Whitney U test for parametric and non-parametric data. Categorical data were compared by χ^2^ Test. Survivals were evaluated by Kaplan-Meier analysis and compared by log-rank test. Multivariate analyses of clinical and genetic parameters were analyzed by Cox-regression. P-values of <0·05 were considered statistically significant.

*References*

1. Lam SSY, et al. Homoharringtonine (omacetaxine mepesuccinate) as an adjunct for FLT3-ITD acute myeloid leukemia. Sci Transl Med. 2016;8(359): 359ra129.

2. Man CH, et al. Sorafenib treatment of FLT3-ITD(+) acute myeloid leukemia: favorable initial outcome and mechanisms of subsequent nonresponsiveness associated with the emergence of a D835 mutation. Blood 2012;119(22):5133–43.

3. Zhang C, et al. Sorafenib and omacetaxine mepesuccinate as a safe and effective treatment for acute myeloid leukemia carrying internal tandem duplication of Fms-like tyrosine kinase 3. Cancer. 2020;126(2):344–53.

4. Cibulskis K, et al. Sensitive detection of somatic point mutations in impure and heterogeneous cancer samples. Nat Biotechnol. 2013;31(3):213–9.

5. Au CH, Wa A, Ho DN, Chan TL, Ma ESK. Clinical evaluation of panel testing by next-generation sequencing (NGS) for gene mutations in myeloid neoplasms. Diagn Pathol 2016;11(1):1–12.

6. Landrum MJ, et al. ClinVar: Improving access to variant interpretations and supporting evidence. Nucleic Acids Res. 2018;46(D1):D1062–7.

7. Tate JG, et al. COSMIC: The Catalogue Of Somatic Mutations In Cancer. Nucleic Acids Res. 2019;47:D941–7.

8. de Wreede LC, Fiocco M, Putter H. mstate : An R Package for the Analysis of Competing Risks and Multi-State Models. J Stat Softw 2011;**38**(7):1–30.

9. https://cran.r-project.org/web/packages/survival/vignettes/ concordance.pdf. Date of access: 20th June, 2020.

10. Harrell FE Jr, Lee KL, Mark DB. Multivariable prognostic models: issues in developing models, evaluating assumptions and adequacy, and measuring and reducing errors. Stat Med. 1996;**15**(4):361-87.

**Legends to Supplemental Figures**

Supplemental Figure S1. Multi-stage prediction model based on the present cohort. Clinicopathologic features, genetic mutations and outcomes (remission, progression, death) from the time of diagnosis to death were evaluated. In this model, patients might transit through four disease stages: diagnosis, CR1, first relapse (R1) and death, with CR1 and R1 being intermediate states. The multi-stage model predicted the probability and duration of each transition (T1, 2, 3, 4 and 5). Patient information in each state was used independently to train the model using multivariate Cox regression. Figure 1A showed the number of patients for the training set. In a new patient, these estimated hazard ratios were then used to predict the probability and duration of each transition (T1, 2, 3, 4 and 5). Age, gender, presenting WCC and mutations of *NPM1*, *DNMT3A* and *FLT3*-ITD were defined as predictors of all transitions. Allo-HSCT was defined as predictor of T3-5. To compare the prediction power of this model with that of ELN2017 risk stratification, the latter was used to define patients into favorable, intermediate and unfavorable risk groups as predictors for all transitions.

Supplemental Figure S2. Variant allelic frequency (VAF) of different gene mutations. Each dot represented datum from individual patients.

Supplemental Figure S3. Effects of age and presenting white cell counts (WCC) on leukemia-free (LFS), Overall (OS) and Event-Free survival (EFS). There was no well defined cut-off of age and presenting WCC with respect to their effects on outcome. Therefore, in univariate and multivariate analyses the two parameters were evaluated as numerical data.

Supplemental Figure S4. Leukemia-free, event-free and overall survival in patients with *DNMT3A* mutation receiving standard (60mg/m^2^) and high dose daunorubicin (90mg/m^2^). The latter negated the adverse prognostic impact of DNMT3A mutation.

Supplemental Figure S5. Leukemia-free survival (LFS) based on *DNMT3A* and *NPM1* mutation and *FLT3*-ITD. Patients were grouped according to these mutations and the impacts of the other two mutations on LFS were examined.

Supplemental Figure S6. Overall survival (OS) based on *DNMT3A* and *NPM1* mutation and *FLT3*-ITD. Patients were grouped according to these mutations and the impacts of the other two mutations on OS were examined.

Supplemental Figure S7. Leukemia-free and overall survival of patients based on 8 different combinations of *DNMT3A* and *NPM1* mutations and *FLT3*-ITD.

Supplemental Figure S8. Effects of *IDH1*R132 mutation on leukemia-free survival and overall survival of patients who carried wildtype *NPM1*, *DNMT3A* and *FLT3* (Category 2) in cytogenetically normal acute myeloid leukemia.

Supplemental Figure S9. Effects of *DNMT3A* mutation on leukemia-free survival (A,C,E) and overall survival (B, D, F) in ELN defined favorable (A,B), intermediate (C,D) and unfavorable (E, F) risk groups.

Supplemental Figure S10. Clonal hierarchy and heterogeneity in cytogenetically normal AML (CN-AML). Specific mutations could exist as dominant or subclone mutations. Only the most common mutations were shown. Definition of dominant and subclone mutations was described in the Supplemental Materials.

Supplemental Figure S11. Occurrence of rare mutations when common or ELN defined mutations were excluded. The y-axis indicated the number of patients carrying these mutations.
