# Supplementary figures and images for: Redefining prognostication of de novo cytogenetically normal acute myeloid leukemia in young adults
Source: Blood Cancer J. 2020 Oct 19;10(10):104. doi: 10.1038/s41408-020-00373-4 (PMC7573626; doi:10.1038/s41408-020-00373-4)

Supplemental Figure S1. Multi-stage prediction model in cytogenetically normal AML

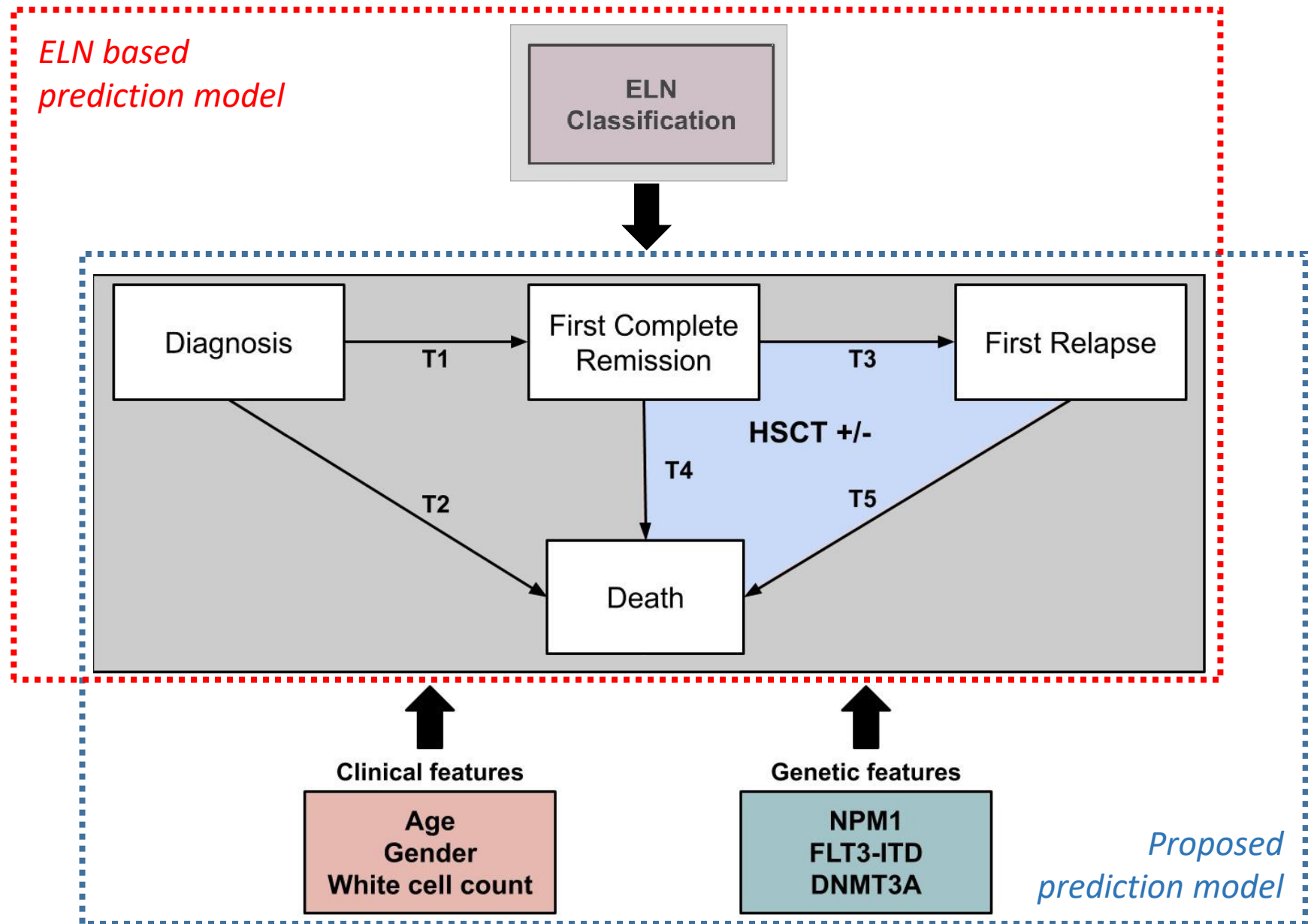

Supplement: Supplementary file 2 — Supplemental figure S1 [file 41408_2020_373_MOESM2_ESM.pdf]

Supplemental Figure S3. Effects of age and presenting WCC on LFS, OS and EFS

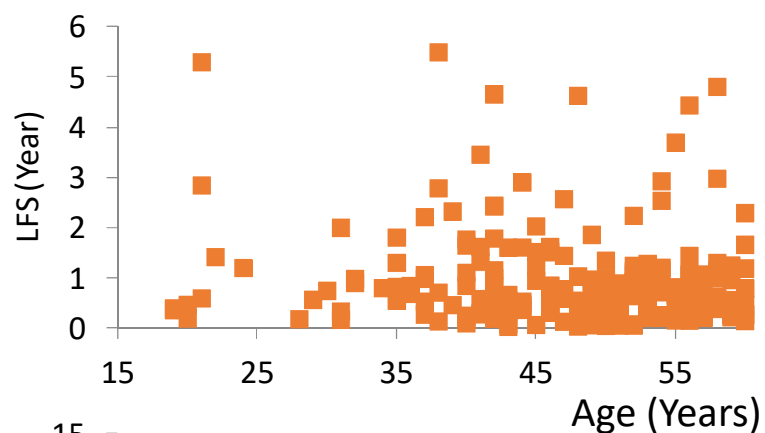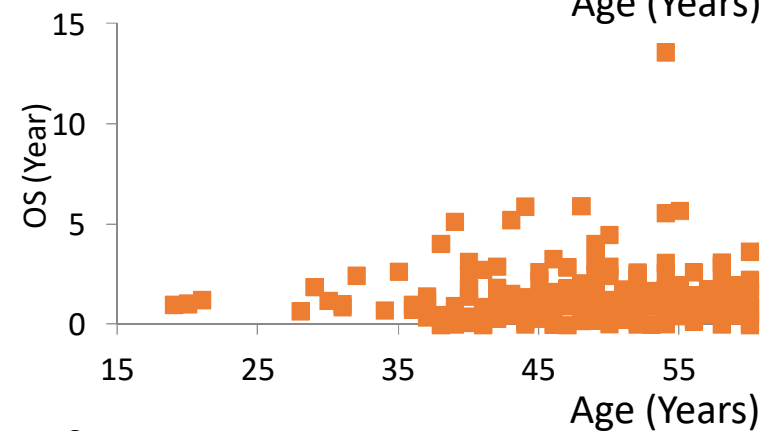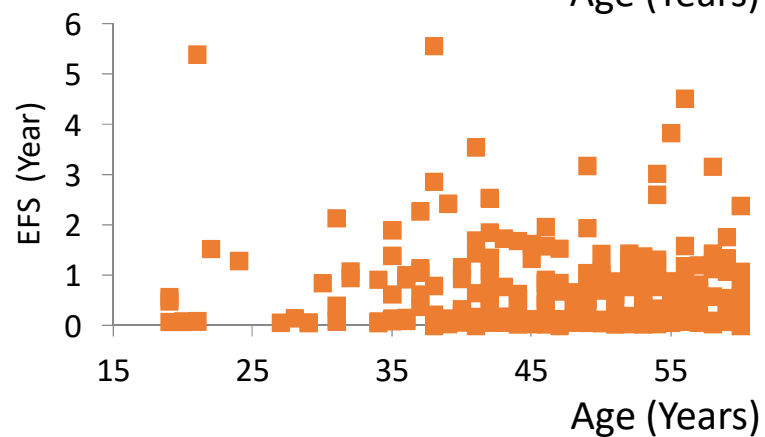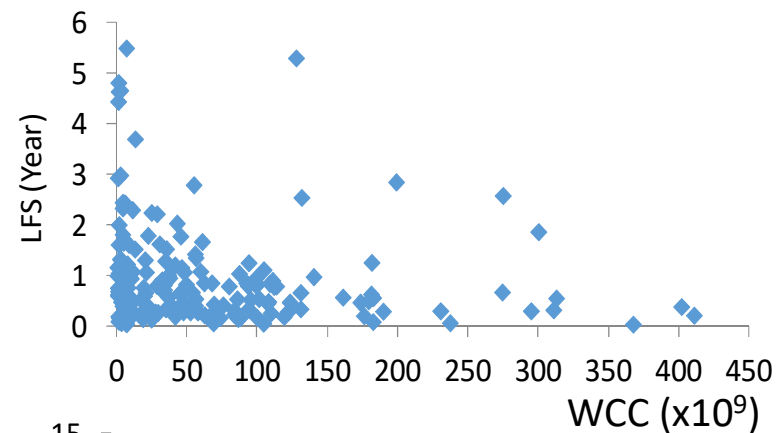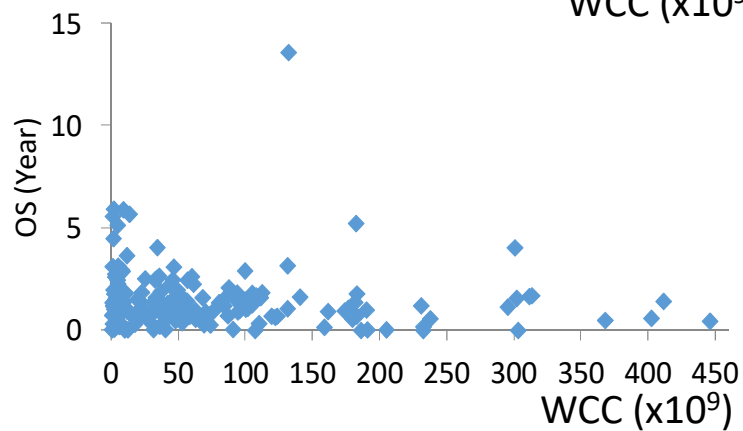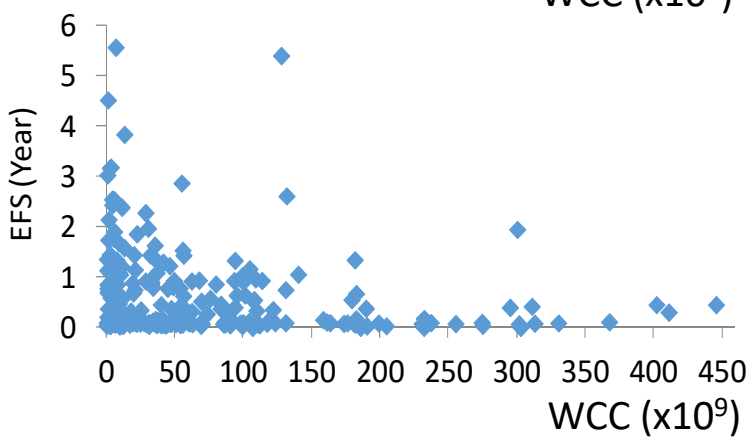

Supplement: Supplementary file 4 — Supplemental figure S3 [file 41408_2020_373_MOESM4_ESM.pdf]

Supplemental Figure S10. Clonal hierarchy and heterogeneity in cytogenetically normal AML.

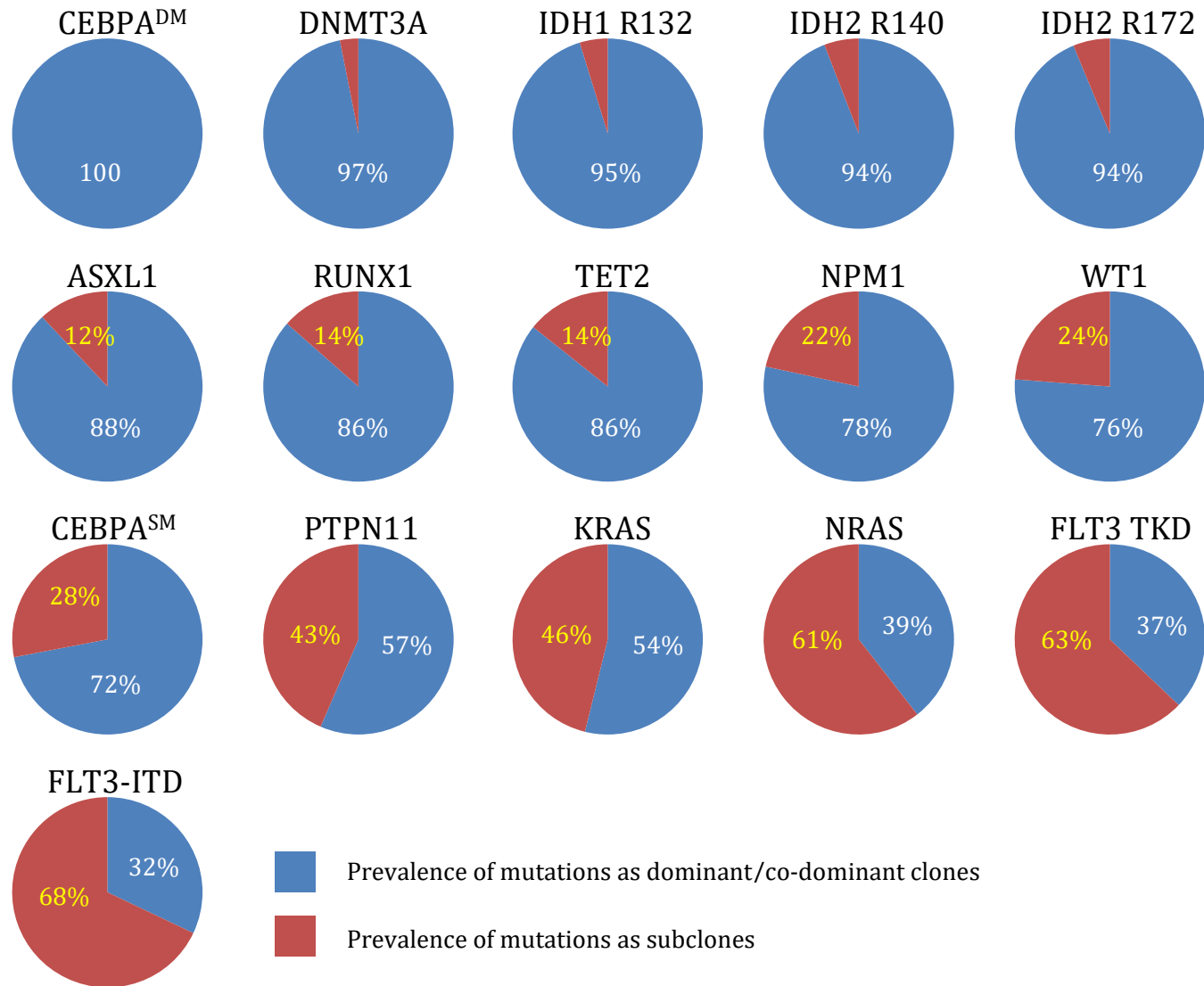

Supplement: Supplementary file 11 — Supplemental figure S10 [file 41408_2020_373_MOESM11_ESM.pdf]
