# Supplementary material for: Redefining prognostication of de novo cytogenetically normal acute myeloid leukemia in young adults
Source: Blood Cancer J. 2020 Oct 19;10(10):104. doi: 10.1038/s41408-020-00373-4 (PMC7573626; doi:10.1038/s41408-020-00373-4)

Supplemental Figure S2. Variant allelic frequency (VAF) of different gene mutations. Each dot represented datum from individual patients.

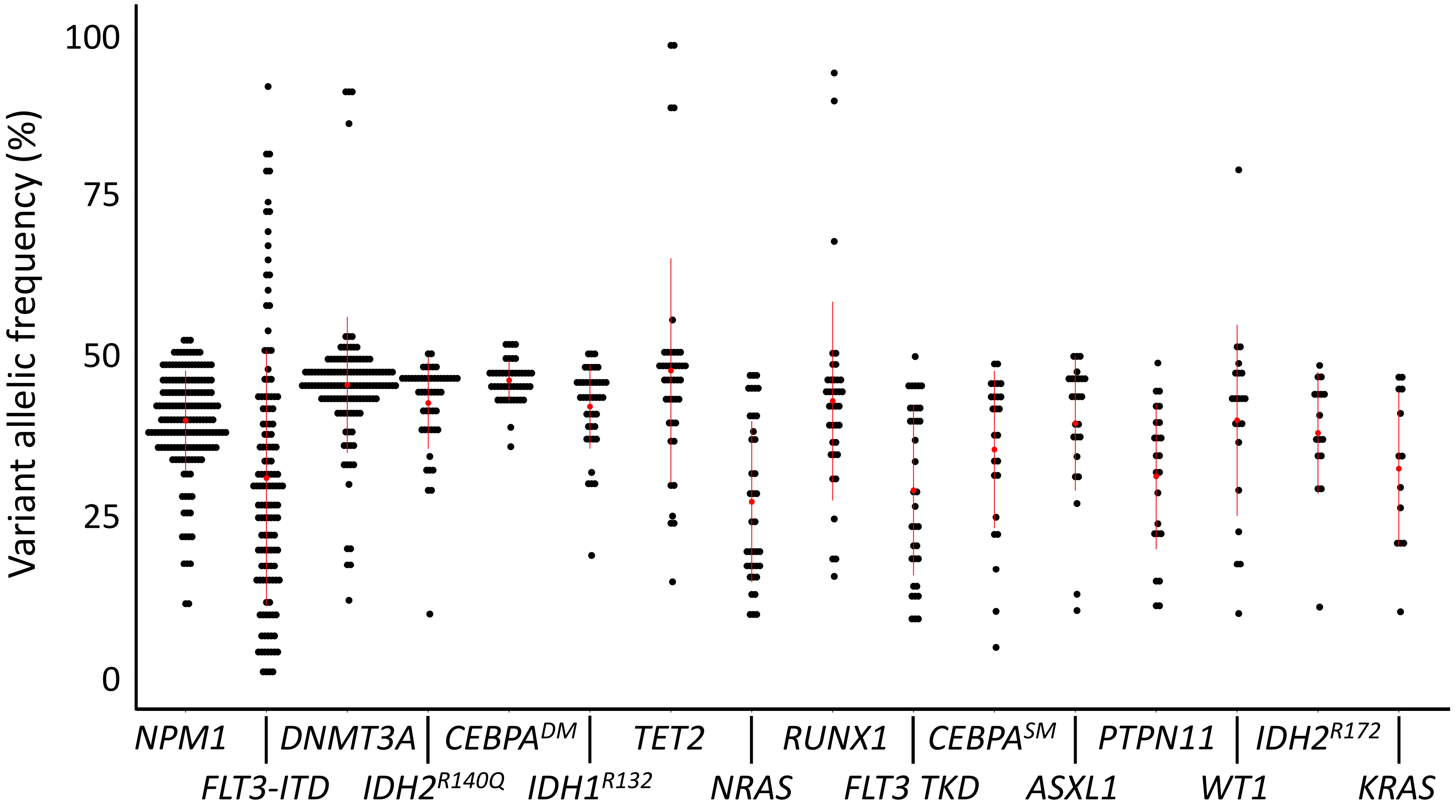

Supplement: Supplementary file 3 — Supplemental figure S2 [file 41408_2020_373_MOESM3_ESM.pdf]
