# Supplementary material for: Redefining prognostication of de novo cytogenetically normal acute myeloid leukemia in young adults
Source: Blood Cancer J. 2020 Oct 19;10(10):104. doi: 10.1038/s41408-020-00373-4 (PMC7573626; doi:10.1038/s41408-020-00373-4)

Supplemental Figure S5. Leukemia-free survival based on *DNMT3A* mutation, *FLT3*-ITD, *NPM1* mutations and their relative impacts

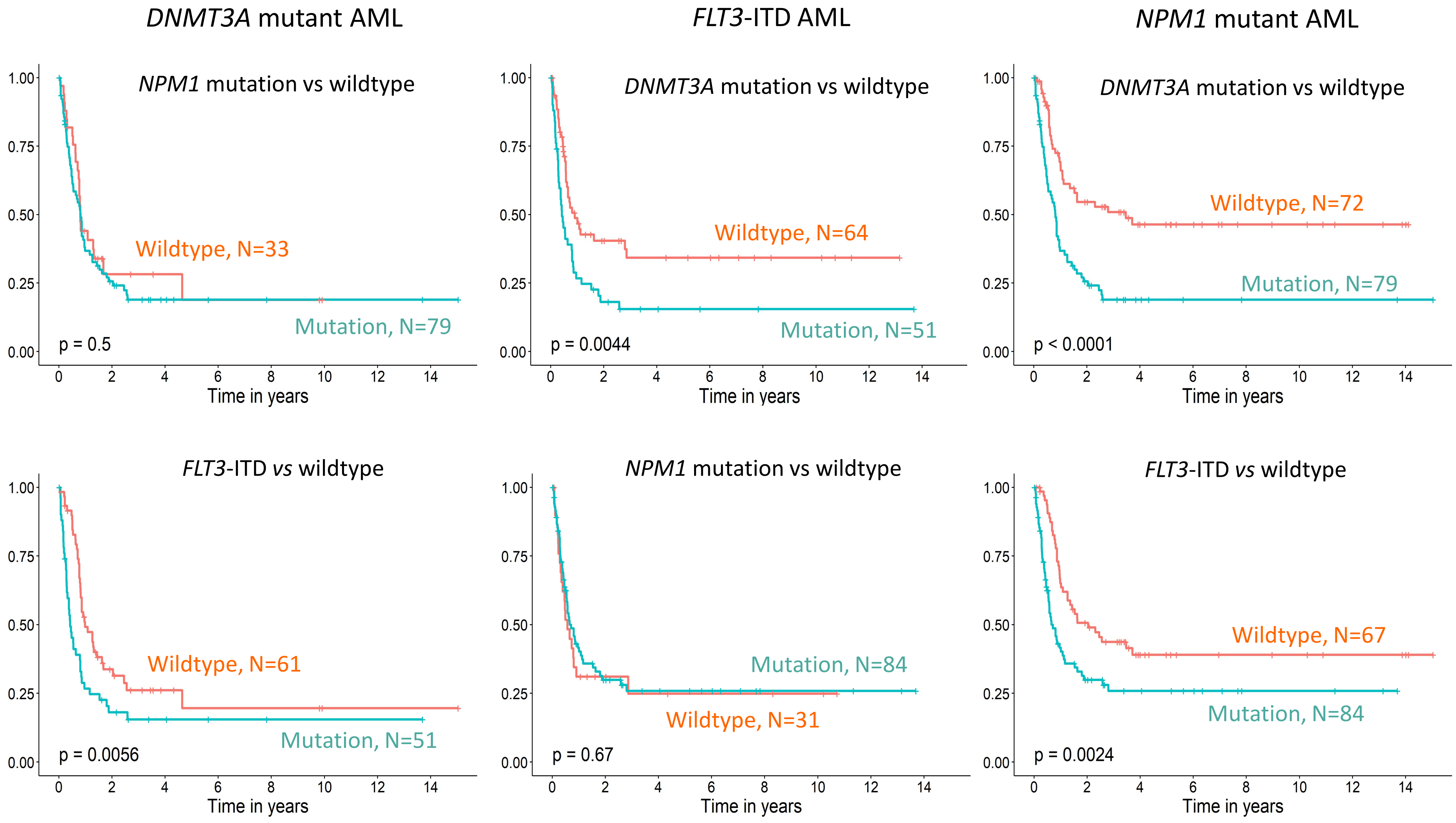

Supplement: Supplementary file 6 — Supplemental figure S5 [file 41408_2020_373_MOESM6_ESM.pdf]
