# Supplementary material for: Redefining prognostication of de novo cytogenetically normal acute myeloid leukemia in young adults
Source: Blood Cancer J. 2020 Oct 19;10(10):104. doi: 10.1038/s41408-020-00373-4 (PMC7573626; doi:10.1038/s41408-020-00373-4)

Supplemental Figure S7. Leukemia free (upper panel) and overall survival (lower panel) based on *DNMT3A* mutation, *FLT3*-ITD and *NPM1* mutations. W: Wildtype; M: mutant or internal tandem duplication for FLT3.

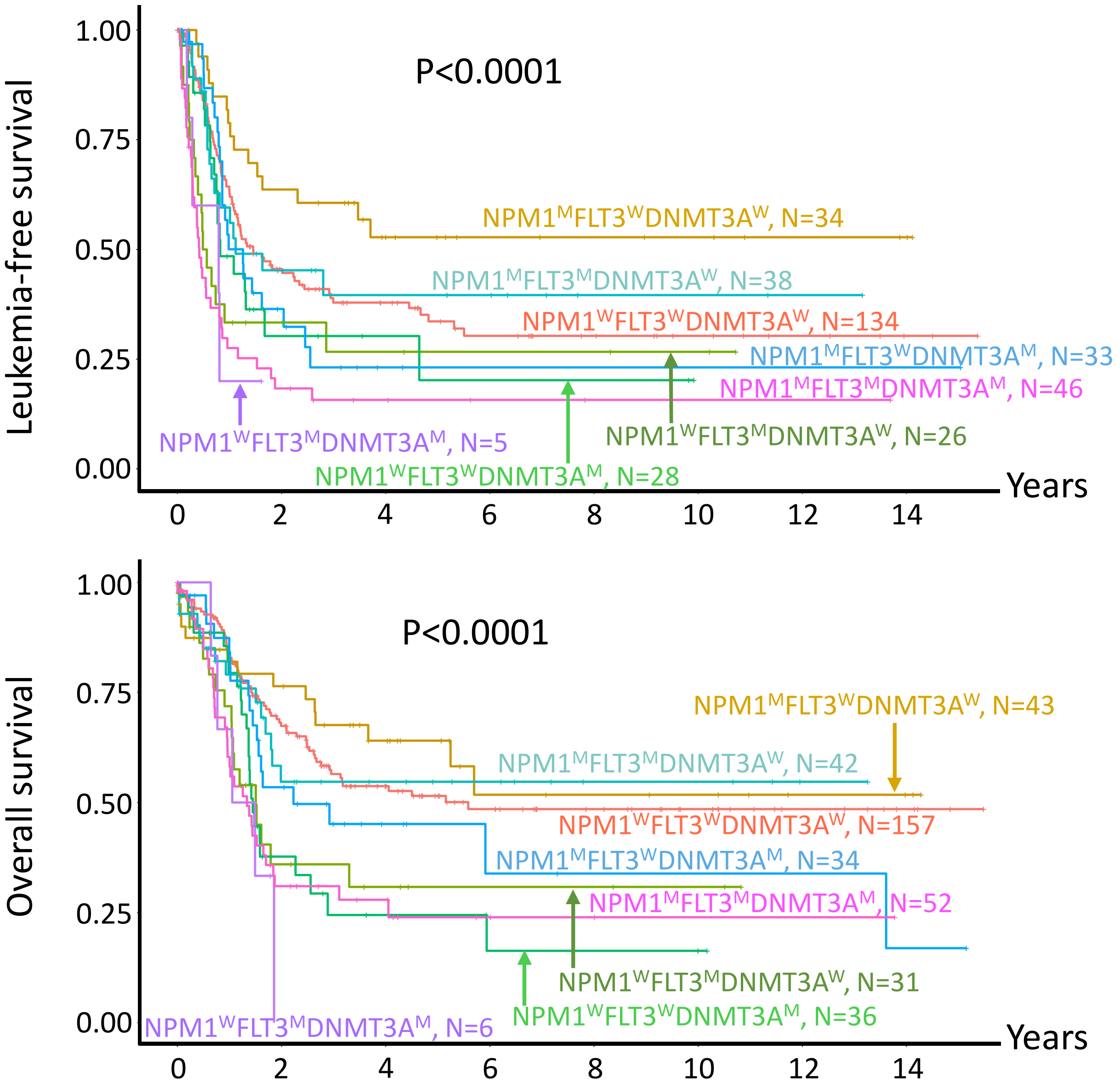

Supplement: Supplementary file 8 — Supplemental figure S7 [file 41408_2020_373_MOESM8_ESM.pdf]
