# Supplementary material for: Redefining prognostication of de novo cytogenetically normal acute myeloid leukemia in young adults
Source: Blood Cancer J. 2020 Oct 19;10(10):104. doi: 10.1038/s41408-020-00373-4 (PMC7573626; doi:10.1038/s41408-020-00373-4)

Supplemental Figure S8. Effects of *IDH1* R132 mutation on leukemia-free survival (A) and overall survival (B) in *NPM1*, *DNMT3A* and *FLT3* wildtype (Category 2).

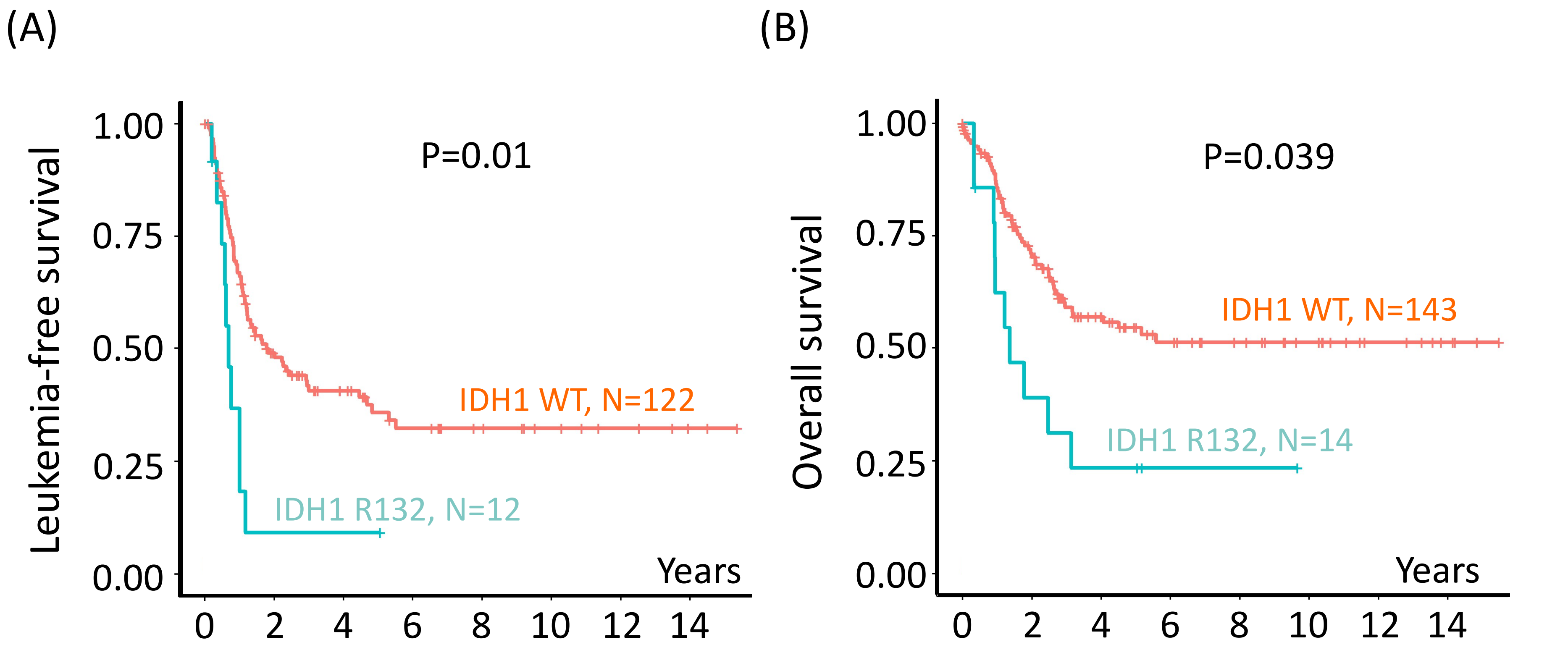

Supplement: Supplementary file 9 — Supplemental figure S8 [file 41408_2020_373_MOESM9_ESM.pdf]
