# Supplementary material for: Redefining prognostication of de novo cytogenetically normal acute myeloid leukemia in young adults
Source: Blood Cancer J. 2020 Oct 19;10(10):104. doi: 10.1038/s41408-020-00373-4 (PMC7573626; doi:10.1038/s41408-020-00373-4)

Supplemental Figure S9. Effects of *DNMT3A* mutation on leukemia-free survival (A,C,E) and overall survival (B,D,F) in ELN defined favorable (A,B), intermediate (C,D) and unfavorable (E,F) risk groups

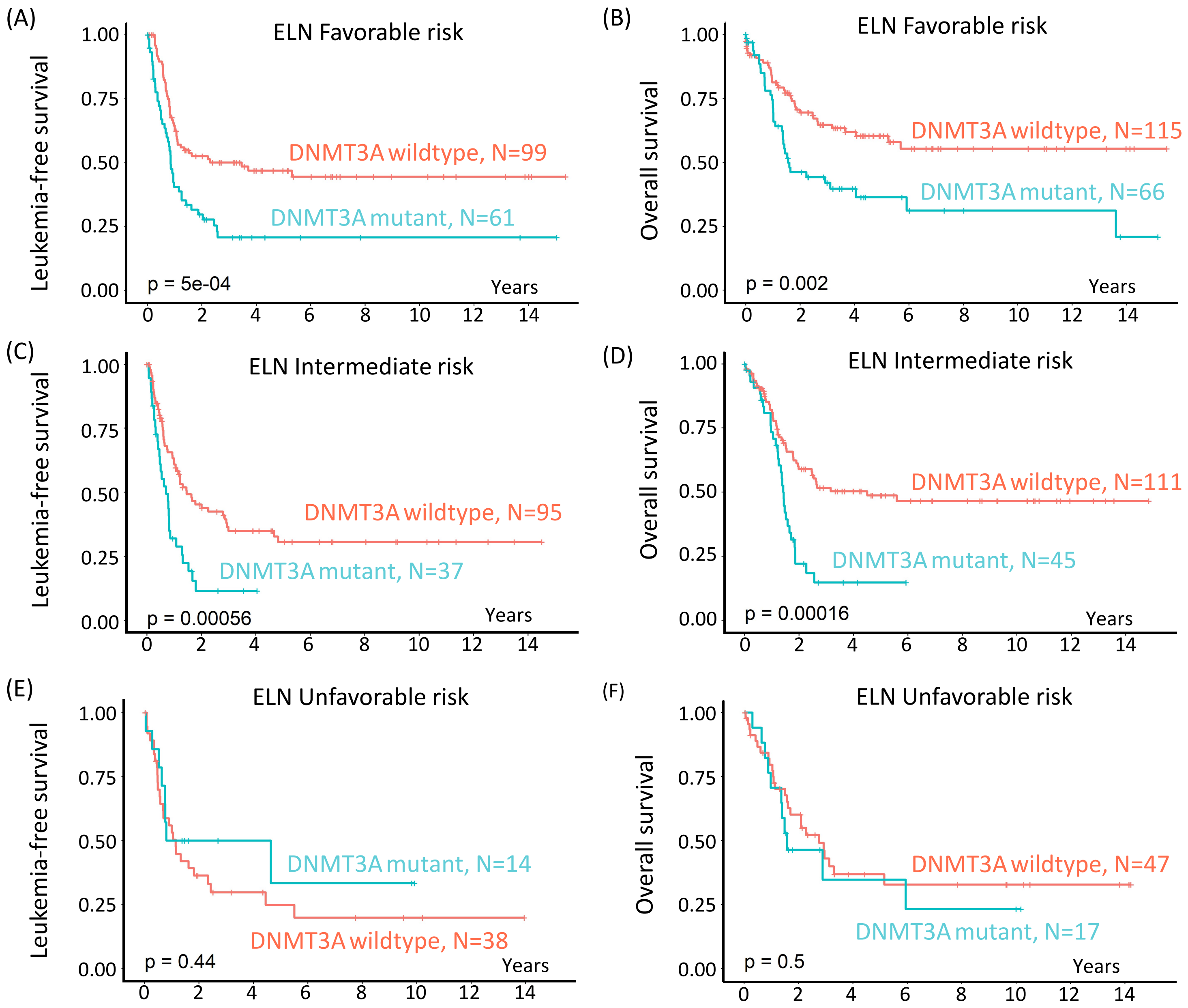

Supplement: Supplementary file 10 — Supplemental figure S9 [file 41408_2020_373_MOESM10_ESM.pdf]
