# Supplementary material for: Redefining prognostication of de novo cytogenetically normal acute myeloid leukemia in young adults
Source: Blood Cancer J. 2020 Oct 19;10(10):104. doi: 10.1038/s41408-020-00373-4 (PMC7573626; doi:10.1038/s41408-020-00373-4)

Supplemental Figure S11. Occurrence of rare mutations when common or ELN defined mutations were excluded.

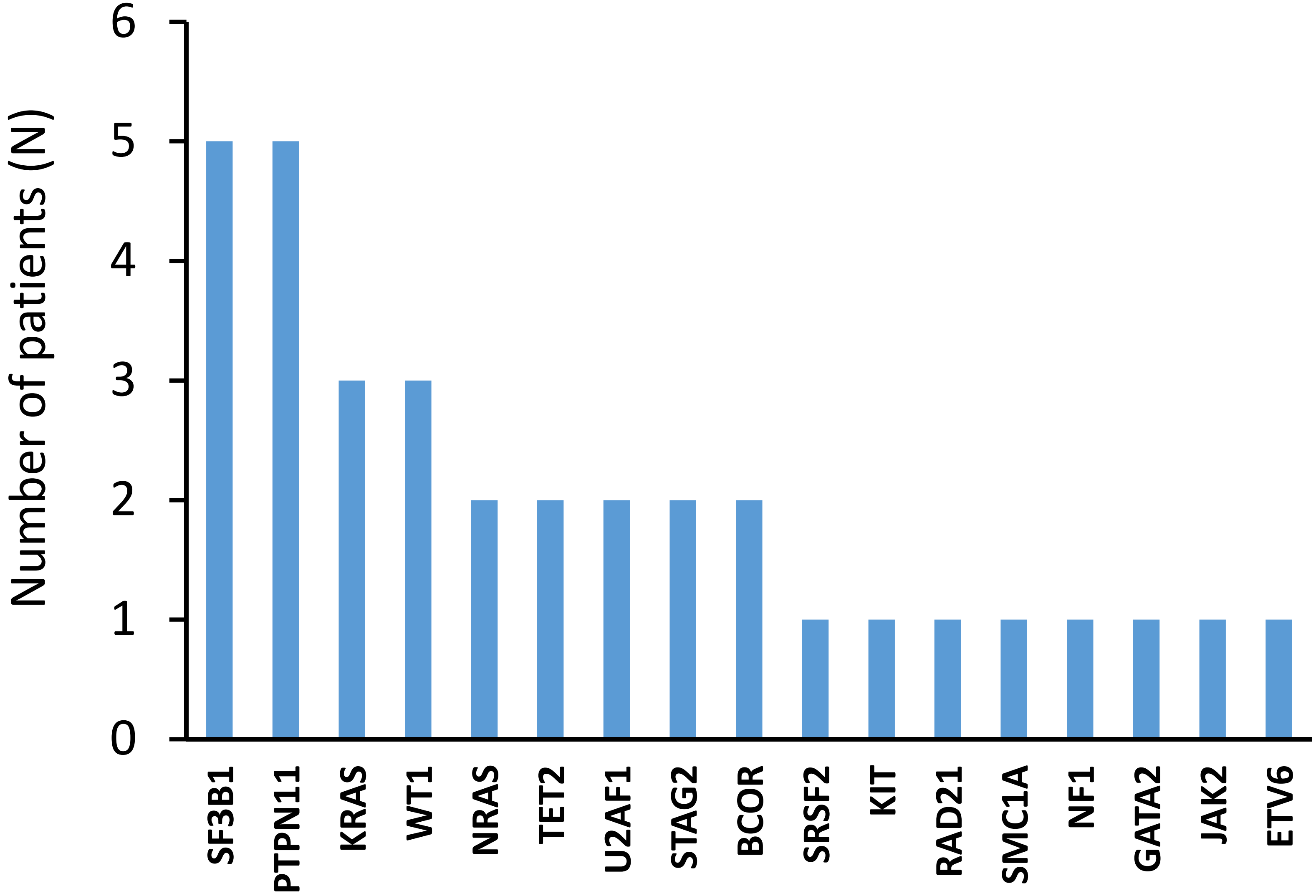

Supplement: Supplementary file 12 — Supplemental figure S11 [file 41408_2020_373_MOESM12_ESM.pdf]
