# Supplementary material for: Redefining prognostication of de novo cytogenetically normal acute myeloid leukemia in young adults
Source: Blood Cancer J. 2020 Oct 19;10(10):104. doi: 10.1038/s41408-020-00373-4 (PMC7573626; doi:10.1038/s41408-020-00373-4)
Supplement: Supplementary file 13 — Supplemental tables [file 41408_2020_373_MOESM13_ESM.docx]

Supplemental Table S1. Clinicopathologic characteristics of *de novo* cytogenetically normal acute myeloid leukemia (CN-AML) patients

| **Features** | **Number** | **Percentage** |
| --- | --- | --- |
| Gender M/F | 199/260 |  |
| Age (Median, Range) (Years) | 49 (18-60) |  |
| Presenting WCC (Median, Range) (x10^9^/L) | 21.6 (0.25-445.6) |  |
| Bone marrow blast % (Median, Range)* | 69.4 (15-99) |  |
| Achieved CR/CRi | 396 | 86.27 |
| After 1 course of induction | 283 | 71.46 |
| After 2 courses of induction | 81 | 20.45 |
| ≥ 3 courses of induction | 32 | 8.08 |
| Received HSCT † | 182 | 39.65 |
| At CR1 | 108 | 59.34 |
| At CR2 | 63 | 34.62 |
| At CR3 | 3 | 1.65 |
| At relapsed state | 8 | 4.40 |

* One patient with BM blasts 15% showed 32% blasts in PB

† One patient received autologous HSCT

Supplemental Table S2. Complete remission (CR) rate of patients who received “7+3” regimen, according to the daunorubicin dosages

| Dose of daunorubicin† | N* | Complete remission (N) | Percentage | P-value |
| --- | --- | --- | --- | --- |
| 60 mg/m^2^ | 199 | 120 | 60.3 | 0.043 |
| 90 mg/m^2^ | 124 | 88 | 70.97 |  |

* The number (N) was limited to those patients with NGS results and documentation of daunorubicin dosages. Among 401 patients with NGS results, 20 patients did not receive induction, 367 patients received “7+3” regimen of whom 44 had no records of daunorubicin dosage. 14 patients received alternative regimens.

† Daily dose for 3 days

Supplementary Table S3. Functions of mutated genes in cytogenetically normal acute myeloid leukemia (CN-AML)

| **DNA methylation** | | | **Nucleophosmin** | | | **Transcription factor** | | | **Chromatin modifiers** | | |
| --- | --- | --- | --- | --- | --- | --- | --- | --- | --- | --- | --- |
| Gene | Count | % | Gene | Count | % | Gene | Count | % | Gene | Count | % |
| DNMT3A | 128 | 31.9 | NPM1 | 171 | 42.6 | CEBPA^DM^ | 46 | 11.5 | ASXL1 | 25 | 6.2 |
| IDH2 R140 | 51 | 12.7 |  |  |  | CEBPA^SM^ | 25 | 6.2 | BCOR | 15 | 3.7 |
| IDH2 R172 | 16 | 4.0 |  |  |  | RUNX1 | 37 | 9.2 | EZH2 | 2 | 0.5 |
| IDH1 | 42 | 10.5 |  |  |  | GATA2 | 13 | 3.2 |  |  |  |
| TET2 | 42 | 10.5 |  |  |  | ETV6 | 3 | 0.8 |  |  |  |
| WT1 | 21 | 5.2 |  |  |  |  |  |  |  |  |  |
| **Activated signaling** | | | **RNA splicing** | | | **Cohesin Complex** | | | **Tumour Suppressor** | | |
| Gene | Count | % | Gene | Count | % | Gene | Count | % | Gene | Count | % |
| FLT3-ITD | 131 | 32.7 | SRSF2 | 17 | 4.2 | STAG2 | 17 | 4.2 | PHF6 | 6 | 1.5 |
| FLT3 TKD | 35 | 8.7 | SF3B1 | 9 | 2.2 | RAD21 | 10 | 2.5 | TP53 | 1 | 0.3 |
| NRAS | 38 | 9.5 | U2AF1 | 5 | 1.3 | SMC1A | 3 | 0.8 |  |  |  |
| KRAS | 13 | 3.2 | ZRSR2 | 1 | 0.2 | SMC3 | 1 | 0.3 |  |  |  |
| PTPN11 | 23 | 5.7 |  |  |  |  |  |  |  |  |  |
| NF1 | 7 | 1.8 |  |  |  |  |  |  |  |  |  |
| KIT | 5 | 1.2 |  |  |  |  |  |  |  |  |  |
| JAK2 | 4 | 1.0 |  |  |  |  |  |  |  |  |  |
| CBL | 3 | 0.8 |  |  |  |  |  |  |  |  |  |

Supplemental Table S4. Complete remission rate after first induction in cytogenetically normal acute myeloid leukemia (CN-AML) carrying recurrent gene mutations. # Fisher’s exact test was used instead because of relatively small sample size.

|  | Rate of complete remission after first induction | | |
| --- | --- | --- | --- |
|  | **Wildtype** | **Mutant** | **P-value** |
| NPM1 | 55.9 | 75.5 | < 0.001 |
| FLT3-ITD | 66.8 | 58.4 | 0.263 |
| DNMT3A | 65.0 | 62.1 | 0.522 |
| IDH2 R140 | 62.9 | 72.3 | 0.218 |
| IDH2 R172 | 65.0 | 40.0 | 0.055 |
| NRAS | 62.9 | 75.0 | 0.329 |
| KRAS | 64.3 | 54.6 | 0.518# |
| IDH1 | 62.9 | 74.7 | 0.344 |
| CEBPA^DM^ | 60.8 | 88.6 | < 0.001 |
| TET2 | 63.8 | 65.9 | 1 |
| RUNX1 | 66.6 | 40.5 | 0.004 |
| FLT3 TKD | 63.7 | 67.7 | 0.811 |
| CEBPA^SM^ | 63.2 | 76.0 | 0.248 |
| WT1 | 65.0 | 47.6 | 0.104 |
| ASXL1 | 66.0 | 36.0 | 0.002 |
| PTPN11 | 64.8 | 52.2 | 0.332 |

Supplemental Table S5A. Impact of individual gene mutations and clinicopathologic parameters on leukemia-free (LFS), event-free (EFS) and overall survival (OS) by univariate analysis.

| **Univariate analysis of individual gene mutations** | | | | | | | | | |
| --- | --- | --- | --- | --- | --- | --- | --- | --- | --- |
| Gene mutations | LFS | | | EFS (CR aft 1st induction) | | | OS | | |
|  | Hazard ratio | 95% CI | p-value | Hazard ratio | 95% CI | p-value | Hazard ratio | 95% CI | p-value |
| NPM1 | 1.03 | 0.78-1.35 | 0.85 | 0.76 | 0.60-0.97 | 0.03 * | 1.06 | 0.79-1.41 | 0.70 |
| FLT3-ITD | 1.68 | 1.27-2.22 | 0.0003 *** | 1.44 | 1.12-1.84 | 0.0039 ** | 1.61 | 1.20-2.16 | 0.002 ** |
| DNMT3A | 1.74 | 1.32-2.30 | <0.0001 *** | 1.37 | 1.07-1.75 | 0.01 * | 1.91 | 1.43-2.55 | <0.0001 *** |
| IDH2 R140 | 1.20 | 0.81-1.77 | 0.37 | 0.88 | 0.61-1.26 | 0.49 | 1.14 | 0.75-1.72 | 0.54 |
| IDH2 R172 | 1.15 | 0.64-2.05 | 0.65 | 1.40 | 0.82-2.40 | 0.22 | 0.82 | 0.40-1.67 | 0.59 |
| NRAS | 1.12 | 0.73-1.73 | 0.60 | 0.82 | 0.55-1.24 | 0.35 | 1.29 | 0.83-2.01 | 0.26 |
| KRAS | 0.96 | 0.42-2.15 | 0.91 | 1.32 | 0.72-2.42 | 0.36 | 1.38 | 0.68-2.79 | 0.38 |
| IDH1 | 1.33 | 0.89-1.97 | 0.17 | 1.02 | 0.70-1.47 | 0.93 | 1.16 | 0.76-1.78 | 0.49 |
| CEBPA^DM^ | 0.80 | 0.52-1.22 | 0.30 | 0.64 | 0.43-0.95 | 0.026 * | 0.55 | 0.33-0.94 | 0.03 * |
| TET2 | 0.64 | 0.40-1.02 | 0.06 | 0.82 | 0.56-1.22 | 0.33 | 0.68 | 0.40-1.13 | 0.14 |
| RUNX1 | 0.93 | 0.57-1.51 | 0.77 | 1.47 | 1.01-2.15 | 0.05 * | 1.21 | 0.76-1.93 | 0.42 |
| FLT3 TKD | 0.86 | 0.54-1.37 | 0.52 | 0.90 | 0.60-1.36 | 0.63 | 0.85 | 0.51-1.42 | 0.54 |
| CEBPA^SM^ | 0.58 | 0.31-1.10 | 0.10 | 0.62 | 0.36-1.06 | 0.08 | 0.50 | 0.24-1.07 | 0.07 |
| WT1 | 1.37 | 0.78-2.40 | 0.26 | 1.32 | 0.79-2.18 | 0.29 | 1.46 | 0.83-2.56 | 0.19 |
| ASXL1 | 1.21 | 0.72-2.05 | 0.47 | 1.77 | 1.16-2.72 | 0.009 ** | 1.08 | 0.64-1.83 | 0.78 |
| PTPN11 | 0.71 | 0.36-1.38 | 0.31 | 0.85 | 0.50-1.45 | 0.55 | 1.0640 | 0.58-1.96 | 0.84 |
| Age | 1.01 | 0.997-1.03 | 0.12 | 1.01 | 0.997-1.02 | 0.14 | 1.02 | 1.01-1.04 | 0.003 ** |
| WCC | 1.003 | 1.001-1.005 | 0.001** | 1.003 | 1.002-1.004 | <0.0001 *** | 1.003 | 1.002-1.005 | <0.0001 *** |
| Dauno dose (90mg/m^2^) | 0.73 | 0.53-0.998 | 0.049 * | 0.73 | 0.55-0.96 | 0.02 * | 0.70 | 0.49-0.98 | 0.04 * |
| CR/CRi | NA | NA | NA | 0.25 | 0.17-0.35 | <0.0001 *** | 0.13 | 0.09-0.19 | <0.0001 *** |
| HSCT at CR1 | 0.26 | 0.18-0.38 | <0.0001 *** | 0.71 | 0.53-0.97 | 0.03 * | 0.33 | 0.219-0.52 | <0.0001 *** |

Supplemental Table S5B. Impact of individual gene mutations and clinicopathologic parameters on leukemia-free (LFS), event-free (EFS) and overall survival (OS) by multivariate analyses.

| **Multivariate analysis on individual gene mutations** | | | | | | | | | |
| --- | --- | --- | --- | --- | --- | --- | --- | --- | --- |
| Gene mutations | LFS | | | EFS (CR aft 1st induction) | | | OS | | |
|  | Hazard ratio | 95% CI | p-value | Hazard ratio | 95% CI | p-value | Hazard ratio | 95% CI | p-value |
| NPM1 | 0.44 | 0.28-0.701 | 0.0004 *** | 0.47 | 0.32-0.70 | 0.0002 *** | 0.67 | 0.41-1.10 | 0.11 |
| FLT3-ITD | 2.76 | 1.79-4.28 | <0.0001 *** | 2.03 | 1.40-2.95 | 0.0002 *** | 2.13 | 1.34-3.38 | 0.001 ** |
| DNMT3A | 2.05 | 1.40-3.02 | 0.0003 *** | 1.49 | 1.08-2.07 | 0.02 * | 2.02 | 1.36-3.00 | 0.0005 *** |
| IDH2 R140 | 0.83 | 0.52-1.34 | 0.45 | 0.89 | 0.57-1.38 | 0.60 | 0.79 | 0.47-1.32 | 0.37 |
| IDH2 R172 | 0.42 | 0.18-0.98 | 0.04 * | 0.73 | 0.34-1.59 | 0.42 | 0.75 | 0.28-1.98 | 0.56 |
| NRAS | 1.01 | 0.59-1.73 | 0.981 | 0.78 | 0.47-1.30 | 0.34 | 1.61 | 0.89-2.89 | 0.11 |
| KRAS | 0.54 | 0.18-1.58 | 0.26 | 2.08 | 0.94-4.62 | 0.07 | 1.10 | 0.40-3.04 | 0.85 |
| IDH1 | 1.12 | 0.67-1.86 | 0.67 | 0.84 | 0.51-1.37 | 0.49 | 1.12 | 0.65-1.93 | 0.68 |
| CEBPA^DM^ | 0.68 | 0.38-1.22 | 0.20 | 0.55 | 0.32-0.94 | 0.03 * | 0.60 | 0.29-1.25 | 0.17 |
| TET2 | 0.62 | 0.36-1.08 | 0.09 | 0.79 | 0.50-1.26 | 0.32 | 0.91 | 0.50-1.67 | 0.76 |
| RUNX1 | 0.55 | 0.29-1.03 | 0.06 | 0.92 | 0.56-1.50 | 0.73 | 1.06 | 0.58-1.96 | 0.84 |
| FLT3 TKD | 0.81 | 0.44-1.48 | 0.49 | 1.02 | 0.60-1.74 | 0.94 | 0.80 | 0.42-1.54 | 0.51 |
| CEBPA^SM^ | 0.51 | 0.21-1.22 | 0.13 | 0.56 | 0.27-1.17 | 0.12 | 0.47 | 0.144-1.53 | 0.21 |
| WT1 | 1.17 | 0.57-2.39 | 0.66 | 0.86 | 0.44-1.70 | 0.67 | 1.18 | 0.54-2.587 | 0.68 |
| ASXL1 | 2.14 | 1.06-4.31 | 0.03 * | 1.74 | 0.98-3.07 | 0.064 | 0.75 | 0.36-1.563 | 0.43 |
| PTPN11 | 0.61 | 0.27-1.41 | 0.25 | 0.93 | 0.48-1.81 | 0.84 | 1.34 | 0.63-2.90 | 0.46 |
| Age | 1.02 | 1.001-1.04 | 0.04 * | 1.01 | 0.99-1.02 | 0.25 | 1.02 | 1.00-1.04 | 0.09 |
| WCC | 1.004 | 1.001-1.01 | 0.002 ** | 1.0016 | 1.00-1.003 | 0.08 | 1.002 | 1.00-1.004 | 0.08 |
| Dauno dose (90mg/m^2^) | 0.72 | 0.51-1.02 | 0.07 | 0.72 | 0.54-0.97 | 0.03 * | 0.79 | 0.54-1.14 | 0.21 |
| CR/CRi | NA | NA | NA | 0.32 | 0.19-0.54 | <0.0001 *** | 0.16 | 0.09-0.30 | <0.0001 *** |
| HSCT at CR1 | 0.10 | 0.06-0.17 | <0.0001 *** | 0.50 | 0.35-0.73 | 0.0003 *** | 0.22 | 0.12-0.38 | <0.0001 *** |

* ≤ 0.05, ** ≤ 0.01, *** ≤ 0.001, NA Not applicable.

Supplemental Table S6. Complete remission rate to first induction in AML subgroups based on recurrent mutations of *NPM1*, *DNMT3A* and *FLT3*-ITD.

| Categories | N | CR after 1^st^ course (N/%) | P-value | Median LFS (months) | Median EFS (months) | Median OS (months) |
| --- | --- | --- | --- | --- | --- | --- |
| 1: *NPM1*^M^/*FLT3*^W^/*DNMT3A*^W^ | 43 | 30/81.1 | 0.07 | NR | 42.7 | 42.7 |
| 2: *NPM1*^W^/*FLT3*^W^/*DNMT3A*^W^ | 157 | 98/65.3 | 0.65 | 17.5 | 10.3 | 17.5 |
| 3: *NPM1*^M^/*FLT3*^M^/*DNMT3A*^W^ | 42 | 27/69.2 | 0.64 | 13.5 | 9.4 | 14.0 |
| 4: *NPM1*^W^/*FLT3*^M^/*DNMT3A*^W^ | 31 | 12/38.7 | 0.01 | 6.4 | 1.2 | 9.0 |
| 5: *NPM1*^M/W^/*FLT3*^M/W^/*DNMT3A*^M^ | 128 | 77/62.1 | 0. 52 | 9.7 | 5.9 | 11.3 |

W: Wildtype. M: Mutation or Internal Tandem Duplication for FLT3; NR: Not reach. CR: Complete remission; CRi: CR with incomplete hematologic recovery; LFS: Leukemia-free survival; EFS: Event-free survival; OS: Overall survival.

Supplemental Table S7. Subgroup analyses.

(A) Subgroup analysis of leukemia-free survival

| Genes | Category | 1 | 2 | 3 | 4 | 5 |
| --- | --- | --- | --- | --- | --- | --- |
| RAS | Mutant | 6 | 18 | 16 | 2 | 1 |
|  | Wildtype | 28 | 116 | 96 | 24 | 37 |
|  | P-value | 0.717† | 0.98 | 0.98 | NA# | NA# |
| TET2 | Mutant | 7 | 12 | 7 | 1 | 11 |
|  | Wildtype | 27 | 122 | 31 | 25 | 101 |
|  | P-value | 0.95† | 0.95 | 0.4† | NA# | 0.4 |
| RUNX1 | Mutant | NA | 14 | 2 | 7 | 8 |
|  | Wildtype | NA | 120 | 36 | 19 | 104 |
|  | P-value | NA | 0.4 | NA# | 0.008**† | 0.039*† |
| IDH2 R140Q and R172K | Mutant | 7 | 14 | 15 | 1 | 21 |
|  | Wildtype | 27 | 120 | 23 | 25 | 91 |
|  | P-value | 0.59† | 0.95† | 0.59 | NA# | 0.59 |
| IDH1 R132 | Mutant | 6 | 12 | 6 | 1 | 12 |
|  | Wildtype | 28 | 122 | 32 | 25 | 99 |
|  | P-value | 0.81† | 0.01* | 0.56† | NA# | 0.13 |
|  |  |  |  |  |  |  |

(B) Subgroup analysis of overall survival

| Genes | Category | 1 | 2 | 3 | 4 | 5 |
| --- | --- | --- | --- | --- | --- | --- |
| RAS | Mutant | 10 | 20 | 16 | 2 | 1 |
|  | Wildtype | 33 | 137 | 112 | 29 | 41 |
|  | P-value | 0.51 | 0.51 | 0.58 | NA# | NA# |
| TET2 | Mutant | 9 | 13 | 7 | 1 | 12 |
|  | Wildtype | 34 | 144 | 35 | 30 | 116 |
|  | P-value | 0.99† | 0.99 | 0.53† | NA# | 0.53 |
| RUNX1 | Mutant | NA | 16 | 2 | 9 | 10 |
|  | Wildtype | NA | 141 | 40 | 22 | 118 |
|  | P-value | NA | 0.96 | NA# | 0.03*† | 0.052 |
| IDH2 R140Q and R172K | Mutant | 9 | 16 | 17 | 1 | 24 |
|  | Wildtype | 34 | 141 | 25 | 30 | 104 |
|  | P-value | 0.53† | 0.53 | 0.53 | NA# | 0.53 |
| IDH1 R132 | Mutant | 7 | 14 | 7 | 1 | 13 |
|  | Wildtype | 36 | 143 | 35 | 30 | 115 |
|  | P-value | 0.78† | 0.04* | 0.34† | NA# | 0.34 |
|  |  |  |  |  |  |  |

* ≤ 0.05, ** ≤ 0.01, *** ≤ 0.001, NA Not applicable.

#: Survival analysis not performed as the number of mutation positive patients in each subgroup is too small

†: Mutation positive patients less than 10 in each subgroup

Supplemental Table S8. Risk stratification based on ELN 2017 guidelines in cytogenetically normal acute myeloid leukemia (CN-AML)

| Risk groups | Mutations |
| --- | --- |
| 1. Favourable risk | NPM1 mutation without FLT3-ITD or low allelic ratio* of FLT3-ITD  CEBPA^DM^ |
| 2. Intermediate risk | NPM1 mutation with high allelic ratio* of FLT3-ITD  Wildtype NPM1 and FLT3 or low allelic ratio of FLT3-ITD* |
| 3. Poor risk | High allelic ratio of FLT3-ITD without NPM1 mutation;  TP53 mutations;  ASXL1 mutations;  RUNX1 mutations |

* Cut-off of allelic ratio of FLT3-ITD was < 0.5 or ≥ 0.5, equivalent to variant allelic frequency (VAF) of < 0.33 or ≥ 0.33.

Supplementary Table S9. Clinicopathologic and mutation features of 17 patients without recurrent AML mutations.

|  | | | | | | | | Mutations other than the 36 genes in AML panel | | | | | |
| --- | --- | --- | --- | --- | --- | --- | --- | --- | --- | --- | --- | --- | --- |
| UPN | Sex | Age | WCC (x10^9^) | PB blast (%) | BM blast (%) | CR | Induction courses | | Gene | Transcript | | Nucleotide change | Amino acid change |
| 259 | F | 49 | 4.83 | 16 | 33 | Y | 1 | | CUL4A | NM_001008895.4 | | c.71_87del | p.Pro24Argfs*82 |
|  |  |  |  |  |  |  |  |  | IGFN1 | NM_001164586.2 | | c.6083C>A | p.Ser2028* |
|  |  |  |  |  |  |  |  |  | KMT2D | NM_003482.4 | | c.13002_13006del | p.Gln4335Profs*35 |
| 357 | F | 50 | 1.32 | 11 | 58 | Y | 1 | | No mutation found | | | | |
| 359 | M | 44 | 1.20 | 2 | 64.5 | Y | 2 | | No Pan-cancer panel results available | | | | |
| 360 | F | 56 | NA | NA | >90 | Y | 1 | | No mutation found | | | | |
| 373 | F | 52 | 8.09 | 77 | 52 | Y | 1 | | SBDS | NM_016038.4 | | c.184A>T | p.Lys62* |
| 538 | M | 58 | 2.70 | 20 | >90 | Y | 1 | | No mutation found | | | | |
| 586 | F | 47 | 1.70 | NA | NA | Y | 1 | | No mutation found | | | | |
| 594 | M | 60 | 3.70 | 5.0 | 81 | Y | 2 | | No mutation found | | | | |
| 639 | F | 38 | 18.68 | 1 | 28 | Y | 1 | | CCND3 | NM_001760.5 | | c.811dup | p.Arg271Profs*53 |
| 742 | M | 37 | 7.16 | 2 | 22 | Y | 1 | | No mutation found | | | | |
| 878 | F | 48 | 1.35 | occ | 26 | Y | 1 | | No mutation found | | | | |
| 921 | F | 19 | NA | NA | NA | Y | 1 | | RAD54L | NM_001142548.1 | | c.1152_1153insACCAGCATTGTGAATAGGTAATGACCTTAAGCCGAGACGCTGCTGCTAGTGAGGCAGACAGGCAGCTAGGAGAGGAGCGGCTGCGGGAGCTC | p.Cys391* |
| 1122 | F | 55 | 6.40 | 39 | 44 | Y | 1 | | No mutation found | | | | |
| 1346 | F | 40 | 4.80 | 20 | NA | Y | 2 | | No mutation found | | | | |
| 1471 | M | 45 | 44.00 | NA | NA | Y | 2 | | No mutation found | | | | |
| 1970 | M | 54 | 0.86 | 6.0 | 56 | N | N | | No mutation found | | | | |
| 2486 | F | 31 | 1.70 | 1 | 25 | Y | 1 | | No mutation found | | | | |
| 2716 | F | 51 | 0.56 | 4.5 | 20 | Y | 1 | | SETD2 | | NM_014159.7 | c.4419dup | p.Asp1474* |
